# Radiative recombination of confined electrons at the MgZnO/ZnO heterojunction interface

**DOI:** 10.1038/s41598-017-07568-z

**Published:** 2017-08-07

**Authors:** Sumin Choi, David J. Rogers, Eric V. Sandana, Philippe Bove, Ferechteh H. Teherani, Christian Nenstiel, Axel Hoffmann, Ryan McClintock, Manijeh Razeghi, David Look, Angus Gentle, Matthew R. Phillips, Cuong Ton-That

**Affiliations:** 1 School of Mathematical and Physical Science, University of Technology Sydney, Broadway, PO Box 123, NSW 2007 Australia; 2 Nanovation, 8 Route de Chevreuse, 78117 Châteaufort, France; 3 Institut für Festkörperphysik, Technische Universität Berlin, 10623 Berlin, Germany; 4 Center for Quantum Devices, ECE Department, Northwestern University, Evanston, IL 60208 USA; 5 Semiconductor Research Centre, Wright State University, Dayton, OH 45435 USA

## Abstract

We investigate the optical signature of the interface in a single MgZnO/ZnO heterojunction, which exhibits two orders of magnitude lower resistivity and 10 times higher electron mobility compared with the MgZnO/Al_2_O_3_ film grown under the same conditions. These impressive transport properties are attributed to increased mobility of electrons at the MgZnO/ZnO heterojunction interface. Depth-resolved cathodoluminescence and photoluminescence studies reveal a 3.2 eV *H*-band optical emission from the heterointerface, which exhibits excitonic properties and a localization energy of 19.6 meV. The emission is attributed to band-bending due to the polarization discontinuity at the interface, which leads to formation of a triangular quantum well and localized excitons by electrostatic coupling.

## Introduction

Oxide heterojunctions are a subject of strong current interest because they provide a wealth of novel functionalities that can be exploited in a broad range of currently emerging technologies involving ultrafast electronics, high-sensitivity chemical sensors and quantum technology^1–4^. The electrical transport properties of the MgZnO/ZnO interface are particularly exciting following the observation of two dimensional electron gas (2DEG) formation at MgZnO/ZnO heterointerfaces due to a strong built-in potential arising from polarization mismatch^5^. These 2DEG samples show remarkably high electron mobilities (in excess of 10^6^ cm^2^/V·s at 40 mK^6^) and exhibit the fractional quantum Hall effect^5^. While the electronic transport properties of MgZnO/ZnO heterojunctions have been investigated by various groups^7,8^, few studies have focused on its optical properties. To date, only optical investigations of indirect excitons have been carried out in MgZnO/ZnO quantum wells^7,9,10^. However, optical characterization of the MgZnO/ZnO interface using such double heterojunction systems has two significant disadvantages. First, the recombination of electrons from sub-bands in excited quantum wells usually take place because of short recombination lifetimes, leading to exciton blue shift and well size-dependent electron-optic effects^11^. Second, the electron mobility in quantum wells is typically lower than in single heterojunctions.

An optical interface emission (called the *H*-band) has been observed in AlGaAs/GaAs^12^ and AlGaN/GaN^13–15^ single heterostructures at low temperatures (T < 20 K), which has been attributed to radiative recombination between photoexcited holes and electrons confined at the interface. Similarly, due to its large band offset and strong strain induced piezoelectric field, the MgZnO/ZnO interface is expected to produce a 2DEG and an associated *H*-band emission that is more robust at higher temperatures because of the high exciton binding energy in ZnO. In this work, MgZnO/ZnO single heterostructures grown by Pulsed Laser Deposition (PLD) are studied using correlative luminescence and electrical characterization techniques. Notably, depth-resolved cathodoluminescence (CL) spectroscopy was employed to investigate the optical emission phenomenon at the MgZnO/ZnO interface. The optical properties of MgZnO/ZnO core-shell nanowires have been investigated previously by other workers^16,17^. The authors reported an enhancement of the near-band-edge (NBE) luminescence; however, the exact cause of the increase could not be identified unambiguously due to the difficulty of spatially resolving the interface geometry in nanowire core-shell structures. Identification of optical signature for MgZnO/ZnO heterostructures constitutes a seminal step in the understanding of localized excitons at the oxide interface and can have major implications for the interpretation of optical and electrical measurements.

## Results and Discussion

### Enhanced conductivity in the MgZnO/ZnO bilayer

**Figure 1:**
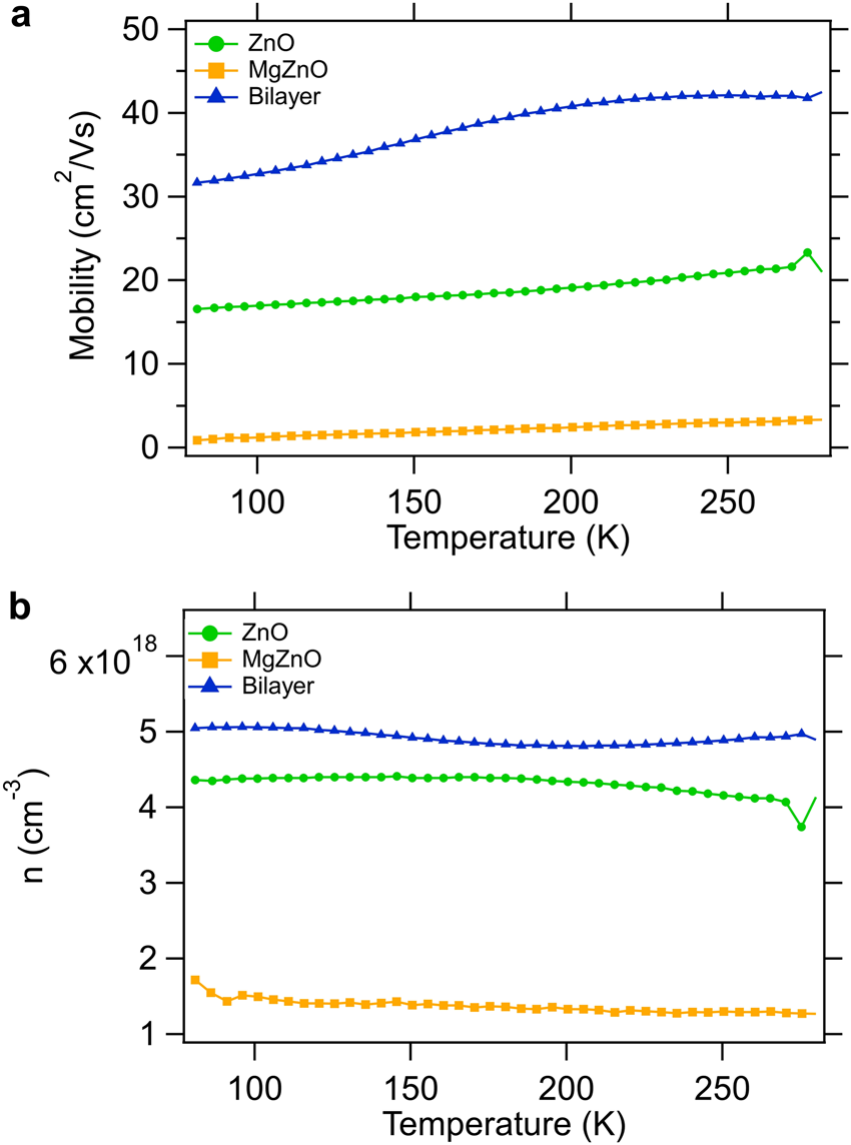
Temperature dependent Hall effect measurements conducted on the MgZnO/ZnO bilayer and constituent single-layer ZnO and MgZnO films on c-sapphire substrate, which yield electron mobility and concentration. At RT, the bilayer exhibits two orders of magnitude lower resistivity and 10 times higher electron mobility compared with the MgZnO/sapphire film grown under the same conditions.

The four-point resistivity of the MgZnO/ZnO/sapphire bilayer is 0.006 Ω.cm, which is more than two orders of magnitude lower than that of the constituent MgZnO film and an order of magnitude lower than that of the constituent ZnO (ρ = 3.0 Ω.cm and 0.1 Ω.cm for the single-layer MgZnO and ZnO films, respectively). This resistivity value is exceptionally low in absolute terms for a ZnO film without intentional shallow donor doping^18^. The reproducibility of the measured resistivity value was confirmed by collecting data at several separate areas of sample with various forward and reverse injection currents and repeating the analysis in three different laboratories. This enhanced conduction is attributed to electron confinement at the smooth MgZnO/ZnO interface. The surface roughness of the ZnO film was measured to be in the range 0.3–0.9 nm over an atomic force microscopy (AFM) scan area of 5 × 5 μm^2^ (shown in Supplementary Fig. S1(b)). Figure 1 shows temperature-dependent Hall mobility and carrier concentration for each of the films. All samples show n-type behavior. For the ZnO single layer, the mobility is about 20 cm^2^/V·s at room temperature (RT) and decreases linearly with temperature to approximately 16 cm^2^/V·s at 77 K. The corresponding carrier concentration rises from a RT value of 4.0 × 10^18^ cm^−3^ to a plateau of 4.4 × 10^18^ cm^−3^ from 180 K to 77 K. For the MgZnO single layer, the RT mobility of 3.5 cm^2^/V·s is significantly lower than that of the ZnO single layer. In a similar manner to the ZnO layer, the MgZnO mobility decreases linearly with temperature to ~0.8 cm^2^/V·s at 77 K. The corresponding carrier concentration is an order of magnitude lower than for the ZnO and rises from a RT value of 1.2 × 10^18^ cm^−3^ to 1.5 × 10^18^ cm^−3^ at 77 K. At 42 cm^2^/V·s, the RT mobility for the MgZnO/ZnO bilayer is significantly higher than for the ZnO or MgZnO single layers. As the temperature decreases, the mobility of the bilayer remains fairly constant down to ~200 K, after which it drops to 31 cm^2^/V·s at 77 K. This temperature-dependent behavior is different from those of the ZnO or MgZnO single layers, which exhibit monotonic decreases in mobility with decreasing temperature. This could be caused by the presence of highly mobile electrons and interface roughness scattering. The carrier concentration of the bilayer is higher than for the ZnO single layer and drops from a RT value of 5.0 × 10^18^ cm^−3^ to a minimum value of 4.8 × 10^18^ cm^−3^ at 180 K.

### Luminescence band originating from the heterostructure interface

**Figure 2:**
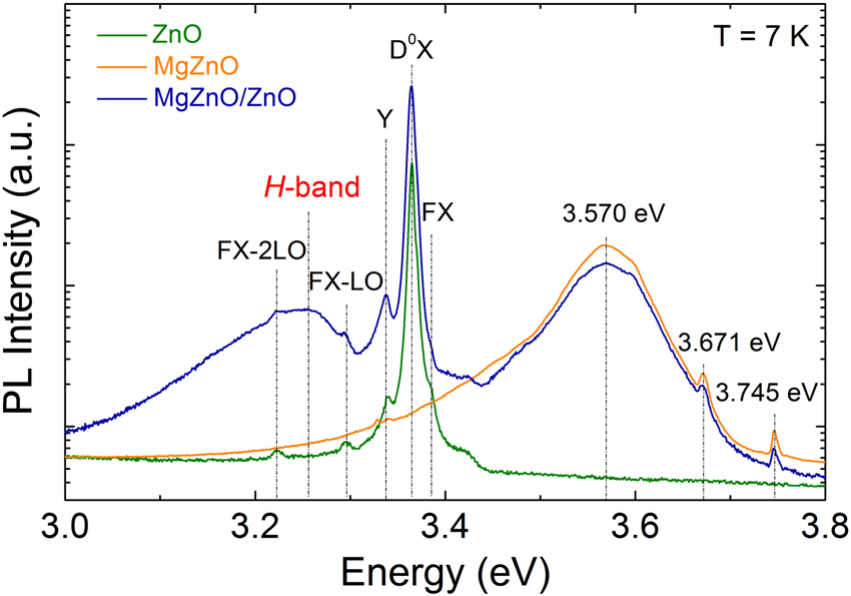
High-resolution PL spectra of three samples of ZnO/c-sapphire, MgZnO/c-sapphire, and MgZnO/ZnO/c-sapphire at 7 K using a 325 nm laser excitation source. The bilayer exhibits a luminescence *H*-band at ~3.2 eV, which is absent in the constituent single-layer films.

Figure 2 shows photoluminescence (PL) spectra for the ZnO, MgZnO, and MgZnO/ZnO samples. The ZnO spectrum exhibits a dominant donor-bound exciton (D^0^X) peak at 3.365 eV, attributed to an I_3_ ionized donor, while a weaker peak on the right shoulder at 3.385 eV is due to free exciton transitions (FX)^19,20^. The peaks at 3.295 eV and 3.221 eV are the first and second order longitudinal optical (LO) phonon replicas of the FX, respectively, with a constant spacing equal to the ZnO LO phonon energy of 72 meV. The presence of the phonon replica peaks indicates high optical quality of the film. The broad NBE emission of the MgZnO and MgZnO/MgO films at 3.570 eV are blue shifted with respect to that of the ZnO due to the enlarged bandgap caused by Mg substitution on Zn sites^21^. The broadening of the MgZnO emission is due to potential fluctuation effects, arising from variation in the Mg composition and lattice disorder in the MgZnO alloy^22^. The absorption edges in RT optical transmission spectra (Supplementary Fig. S3) corresponded to a bandgap energy of 3.61 eV for the MgZnO/ZnO. This corresponds to an Mg content of 16.4 at%^23,24^, which is 2.7 at% more than the nominal composition of the MgZnO PLD target. Such an Mg enhancement compared to the target concentration has been reported elsewhere for PLD growth of MgZnO and was attributed to a difference in the vapor pressures of Mg and Zn^18^. The two sharp peaks at 3.671 eV and 3.743 eV, located at 72 meV and 144 meV from the laser excitation line, are E_1_(LO) and 2E_1_(LO) Raman modes of ZnO. The most significant feature in Fig. 2 is the emergence of a broad emission band at ~3.2 eV (denoted *H*-band) in the MgZnO/ZnO bilayer. This luminescence band is absent in both the single ZnO and MgZnO films, suggesting that it originates from the heterojunction interface.

### Depth-resolved CL microanalysis of the MgZnO/ZnO interface *H*-band

**Figure 3:**
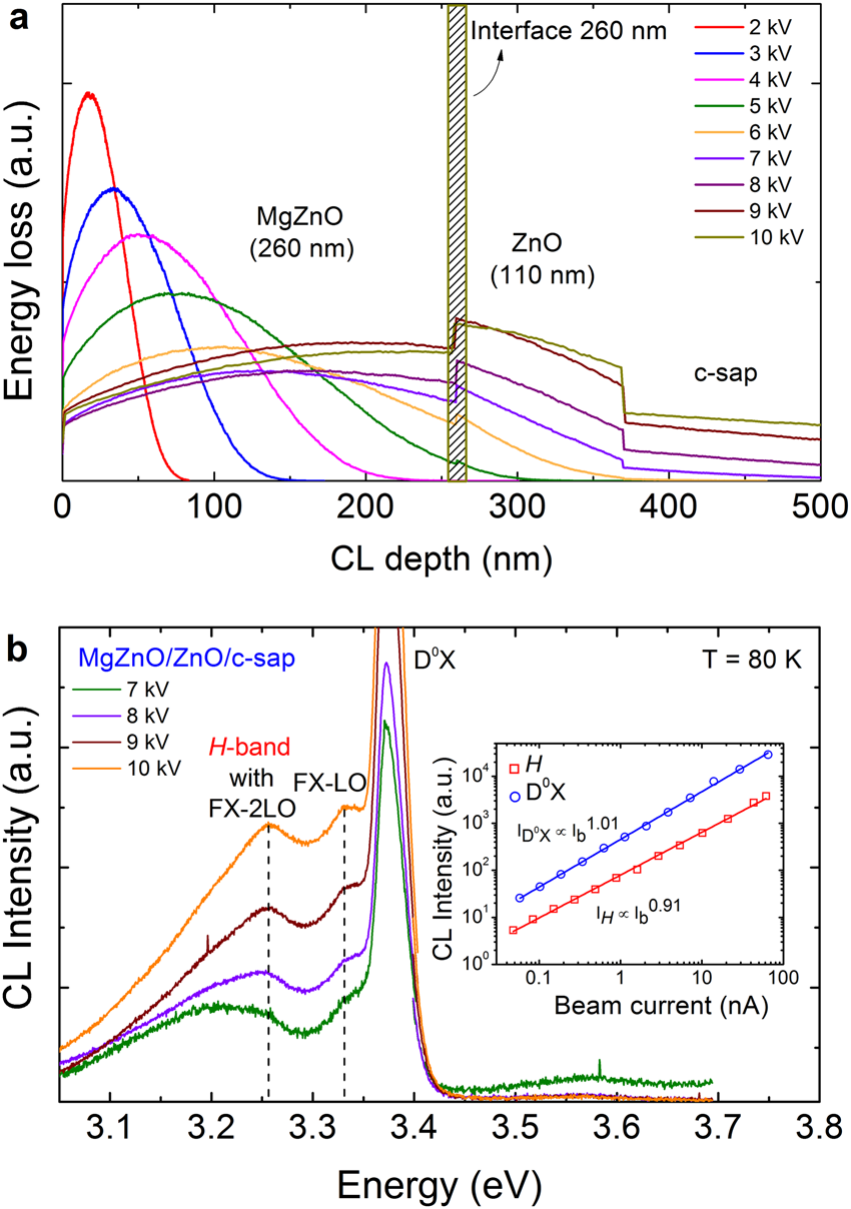
Depth-resolved CL measurements of the MgZnO/ZnO/c-sapphire bilayer. (**a**) CASINO simulated electron energy loss curves for acceleration voltages from 2 kV to 10 kV. The vertical axis corresponds to the percentage of CL generated at that particular depth. (**b**) Depth-resolved CL spectra of the bilayer structure acquired at different acceleration voltages with constant beam power *I*_o_ *V*_o_ = 28 µW. The *H*-band emerges when the electron beam reaches the MgZnO/ZnO interface. Inset shows power density measurements of the MgZnO/ZnO bilayer for D^0^X and *H*-band emissions at 80 K.

To verify the origin of the 3.2 eV band, depth-resolved CL was conducted on each of the three films. In this measurement the electron beam power and hence the electron-hole (e-h) pair generation rate in the sample was kept constant as the CL excitation depth was increased by raising the electron beam energy. The ZnO and MgZnO films exhibit identical CL spectra at all accelerating voltages confirming the in-depth homogeneity of the layer (Supplementary Fig. S3a). To obtain depth-dependent dependences of the CL in the MgZnO/ZnO bilayer, Monte Carlo modeling was carried out using the CASINO simulation package^25^ to determine the electron energy loss profile with the electron beam excitation energy. Figure 3a shows the energy loss profiles of electrons in the bilayer for acceleration voltages between 2 kV and 10 kV obtained from the CASINO simulations. These energy loss profiles are comparable with those obtained for ZnO by other workers^26,27^. The Monte Carlo simulation of the bilayer reveals that the electron beam reaches the 260 nm deep ZnO layer at 5 kV and starts penetrating into the sapphire substrate from 7 kV. The depth-resolved CL measurements of the bilayer (Supplementary Fig. S3b) are consistent with the CASINO modeling results. Figure 3b shows in-depth CL spectra of the bilayer from 7 kV to 10 kV. The bilayer spectra exhibit an additional broad peak positioned at ~3.2 eV, which is not observed in either of the constituent ZnO or MgZnO single layers. The shape of the *H*-band was obtained by subtracting the ZnO luminescence contribution from the bilayer CL emission at 7 kV, yielding a broad spectrum peaked at 3.2 eV (Supplementary Fig. S4). These depth-resolved CL results confirm the *H*-band emission originating from the MgZnO/ZnO heterointerface.

Excitation-power resolved CL analysis of the bilayer at 7 kV was measured by increasing the beam current (*I*_B_) from 0.03 nA to 70 nA and analyzed using the power-law model *I*_CL_ ∝ *I*_B_^*k*^, where *I*_CL_ is the CL intensity and *I*_B_ is beam current^28^. Since the minimum energy of the electron beam necessary to cause knock-on damage is 400 keV for Zn ions and 250 keV for O ions, the electron beam is not expected to damage the bilayer film. The log-log plots based on the power-law model are displayed in the inset of Fig. 3b, which yields the exponent *k* = 1.01 and *k* = 0.91 for the D^0^X and *H*-band peaks, respectively. These results indicate that the *H*-band is not related to a lattice coupled defect, as defect-related emissions in ZnO typically exhibit sublinear dependence on the beam power^29,30^. In addition, as the excitation density is increased, the *H*-band is strongly blue shifted by ~40 meV, while the energy position of the ZnO D^0^X peak remains unchanged (Supplementary Fig. S4). This is likely caused by the band bending field being screened by excess carriers that are generated by the electron beam, providing further support for its assignment of the *H*-band to localized excitons at the MgZnO/ZnO interface.

**Figure 4:**
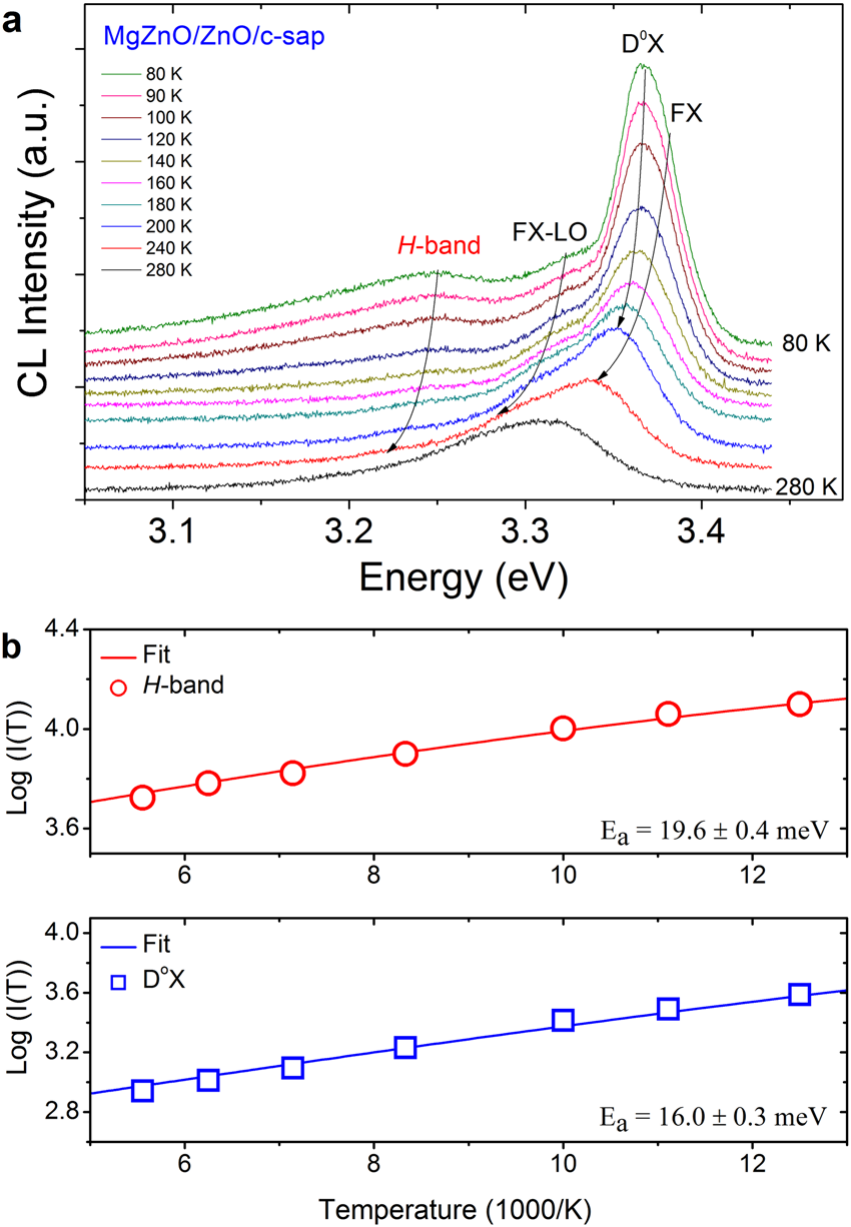
Temperature-resolved CL measurements of the MgZnO/ZnO bilayer. (**a**) Temperature-dependent CL spectra of the bilayer acquired at an acceleration voltage of 7 kV. The *H*-band is identifiable at temperatures up to 160 K. (**b**) Variations of the *H*-band and D^0^X peak intensities as a function of temperature. Activation energies of 19.6 meV and 16.0 meV were determined by fitting these data according to the Arrhenius equation.

Temperature dependent CL was performed to determine the activation energy of the interface emission. Figure 4a shows the temperature-resolved CL spectra of the MgZnO/ZnO bilayer acquired at temperatures from 80 K to 300 K at 7 kV. With increasing temperature, the NBE CL intensity is quenched because of the thermal detachment of bound excitons from shallow donors and the high probability of non-radiative recombination. The *H*-band shows remarkable thermal stability up to 150 K. The activation energies of the D^0^X and *H*-band can be determined according to the Arrhenius law equation:

1 $$I(T)\,=\,\frac{I(0)}{{\rm{1}}+C\,\exp (-{E}_{a}/{k}_{b}T)}$$

where *I*(0) is the luminescence intensity at 0 K, *E*_*a*_ is the activation energy of the emission, and *C* is a scaling factor. Figure 4b shows the D^0^X and *H*-band intensities of the bilayer against temperature; fitting these data to the Arrhenius equation gives *E*_*a*_ = 19.6 ± 0.4 meV for the *H*-band and 16.0 ± 0.3 meV for the D^0^X line. The *E*_a_ value for the D^0^X line is within the range of reported activation energies of bound excitons (10–20 meV)^31^. Because the *E*_a_ of the MgZnO/ZnO *H*-band is significantly greater than that of GaN heterostructures (~10 meV)^32^, the emission from the ZnO is thermally stable at higher temperatures.

### Radiative recombination at the heterointerface

**Figure 5:**
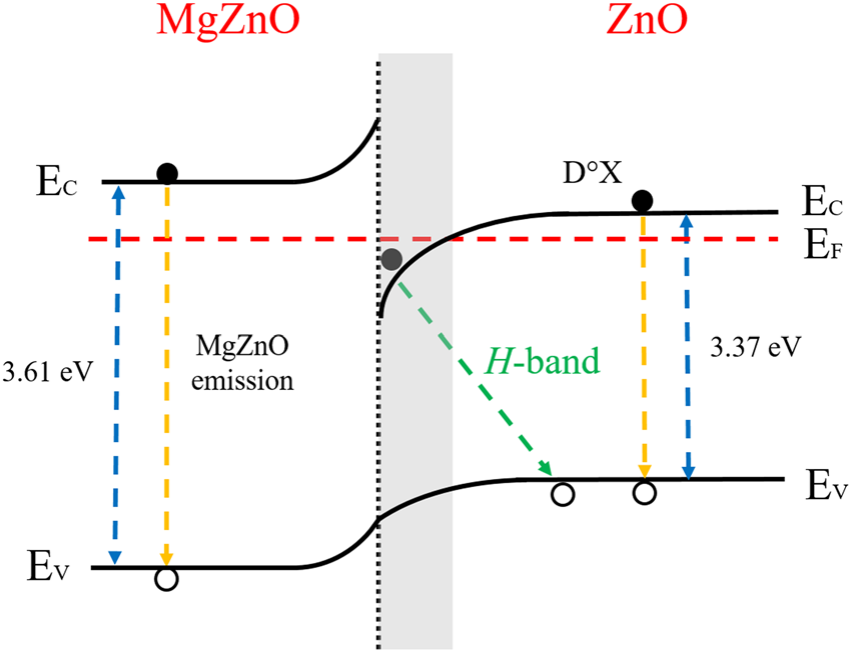
Schematic band diagram of the MgZnO/ZnO heterostructure, showing the recombination channels responsible for the emissions in the bilayer film. Dotted lines represent the excitonic transitions accounting for the observed NBE emissions in ZnO and MgZnO. The *H*-band arises from the recombination of electrons confined within the triangular quantum well with holes located in the flat-band region of the ZnO valence band.

The *H*-band emission in the ZnO/MgZnO heterostructure can be explained by the interface band structure **(**Fig. 5). Using the reported band offset ratio of Δ*E*_v_/Δ*E*_c_ = 0.5^33^, the band-edge discontinuities at the interface can be estimated to be Δ*E*_c_ = 253 meV for the conduction band and Δ*E*_v_ = 127 meV for the valence band. In contrast to AlGaAs/GaAs^12^ modulation-doped heterostructures where a large barrier height at the interface together with the doping level determines the electron distribution at the interface, it is the polarization discontinuity at the MgZnO/ZnO that induces the band bending at its interface, leading to the formation of a triangular electron well^26^, where the electron is confined within the potential well and spatially separated from the hole in the flat-band region of the ZnO valence band. Thus the nergy position of the *H*-band localized exciton can be estimated as ref.^34^:

2 $${E}_{{H}-\mathrm{band}}={E}_{g}-{E}_{e,loc}-{E}_{e,hole}$$

where *E*_g_ is the ZnO band gap, *E*_e,loc_ the localization energy of electrons in the interface potential well and *E*_e,hole_ the energy required to disassociate the hole from the excitonic complex (i.e. the binding energy of the hole). Additionally, it has been pointed out that the activation energy of the *H*-band exciton corresponds to *E*_e,hole_, rather than the localization energy of electron in the potential well^34^. According to these results, we can estimate the binding energy of the hole in the localized exciton to be ~20 meV. From the measured values for the heterostructure, the ground state of electrons in the potential well can be calculated from Eq. (2), *E*_e,loc_ = 110 meV. The polarization sheet charge at the MgZnO/ZnO interface can be estimated as follows^35,36^:

3 $$\sigma ={P}_{sp}(x)-{P}_{sp}(0)-{P}_{pe}(x)$$

where *P*_sp_(*x*) is the spontaneous polarization in the Mg_*x*_Zn_1−*x*_O layer and *P*_pe_ is the piezoelectric polarization, respectively. Analytical solutions for each of the polarization terms have previously been determined by solving Poisson and Schrödinger equations^37^, which yield ${P}_{{sp}}(x)$ = −0.0322 + 0.024*x* C/m^2^ and ${P}_{{pe}}(x)$ = −0.0584*x*. For the MgZnO/ZnO heterostructure in this work (*x* = 0.164), $\sigma =0.0135{C}/{m}^{2}$, which is consistent with the value obtained from the interface scattering model for MgZnO/ZnO heterostructures^38^.

In conclusion, optical studies were conducted on the MgZnO/ZnO heterointerface which showed significantly increased conductivity and electron mobility compared with the constituent layers. Depth-resolved luminescence spectroscopy revealed a radiative recombination *H*-band at 3.2 eV arising from the heterointerface, which was not observed for the constituent single-layer ZnO or MgZnO films. This interfacial *H*-band with a large activation energy of 19.6 meV is stable up to 150 K and attributed to localized excitons that arise due to the presence of confined electrons at the interface as a result of band bending. These findings may provide new opportunities to optically access hidden junctures and to utilize the optical emission arising from recombination of localized excitons in optoelectronic interface devices.

## Methods

MgZnO thin films were grown simultaneously on *c*-sapphire and O-polar ZnO (0001)-coated c-sapphire substrates using pulsed laser deposition (PLD) with a Coherent LPX 100 KrF (248 nm) excimer laser and a commercial sintered MgZnO (13.4 atomic % Mg) target in oxygen, using fabrication conditions described elsewhere^39^. The film thicknesses determined from scanning electron microscope (SEM) cross-sections in an FEI field emission gun XL30S system were 110 nm and 260 nm for the ZnO and MgZnO films, respectively. X-Ray Diffraction (XRD) was performed in a Panalytical MRD Pro system using Cu Kα_1_ radiation. Electrical resistivity was measured using a Signatone co-linear four-point probe system. Temperature dependent Hall measurements were made using a Van der Pauw configuration and indium contacts. The chemical composition of the films was determined using a Zeiss Evo LS15 SEM equipped with a Bruker XFlash 5030 Silicon Drift Detector EDX spectrometer. RT optical transmission studies were performed using an Ocean Optics system comprising a halogen and deuterium lamps plus a Maya spectrometer. CL microanalysis of the films was conducted in an FEI Quanta 200 Environmental SEM equipped with a diamond machined parabolic light collector and a Hamamatsu S7011–1007 CCD spectrometer. Depth-resolved CL measurements were conducted under constant beam power (*I*_o_ *V*_o_ = 28 µW) by increasing the accelerating voltage while adjusting the electron beam current. Under such excitation conditions the e-h pair generation in the sample was kept constant. Additionally, the samples were analyzed by micro-PL spectroscopy in a liquid He bath at 6 K. The samples were excited by 325 nm He-Cd laser line and the emitted light was dispersed by a Spex-1404 double monochromator. The spectra were acquired with an optical power density of 100 kW/cm^2^.

## Electronic supplementary material

Supplementary Information
