# Author Correction: Radiative recombination of confined electrons at the MgZnO/ZnO heterojunction interface

**DOI:** 10.1038/s41598-017-14857-0

**Published:** 2017-11-16

**Authors:** Sumin Choi, David J. Rogers, Eric V. Sandana, Philippe Bove, Ferechteh H. Teherani, Christian Nenstiel, Axel Hoffmann, Ryan McClintock, Manijeh Razeghi, David Look, Angus Gentle, Matthew R. Phillips, Cuong Ton-That

**Affiliations:** 1 School of Mathematical and Physical Science, University of Technology Sydney, Broadway, PO Box 123, NSW 2007 Australia; 2 Nanovation, 8 Route de Chevreuse, 78117 Châteaufort, France; 3 Institut für Festkörperphysik, Technische Universität Berlin, 10623 Berlin, Germany; 4 Center for Quantum Devices, ECE Department, Northwestern University, Evanston, IL 60208 USA; 5 Semiconductor Research Centre, Wright State University, Dayton, OH 45435 USA

*Scientific Reports* 7:7457; doi:10.1038/s41598-017-07568-z; Article published online 07 August 2017

The original version of this Article contained errors in References 1–14, 16–28, 31–32 and 35–38. These errors have now been corrected in the HTML and PDF versions of this paper.

