# Supplementary material for: Radiative recombination of confined electrons at the MgZnO/ZnO heterojunction interface
Source: Sci Rep. 2017 Aug 7;7:7457. doi: 10.1038/s41598-017-07568-z (PMC5547142; doi:10.1038/s41598-017-07568-z)
Supplement: Supplementary file 1 — Supplementary Information [file 41598_2017_7568_MOESM1_ESM.pdf]

Supplementary Information

Radiative recombination of confined electrons at the MgZnO/ZnO heterojunction interface

Sumin Choi[1], David J. Rogers[2], Eric V. Sandana[2], Philippe Bove[2], Ferechteh H. Teherani[2], Christian Nenstiel[3], Axel Hoffmann[3], Ryan McClintock[4], Manijeh Razeghi[4], David Look[5], Angus Gentle[1], Matthew R. Phillips[1], and Cuong Ton-That[1]*

[1]School of Mathematical and Physical Science, University of Technology Sydney, Broadway, PO Box 123, NSW 2007, Australia

[2]Nanovation, 8 Route de Chevreuse, 78117 Châteaufort, France

[3]Institut für Festkörperphysik, Technische Universität Berlin, Germany

[4]Center for Quantum Devices, ECE Department, Northwestern University, Evanston, Il60208, USA

[5]Semiconductor Research Centre, Wright State University, Dayton, OH 45435, USA

* Corresponding author. Email: cuong.ton-that@uts.edu.au

1. Characterization of the MgZnO and ZnO films

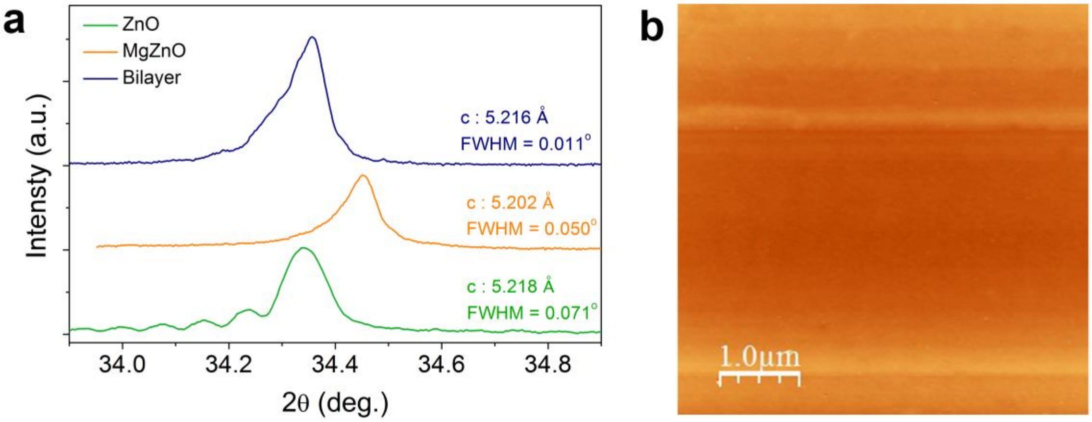

Figure S1. (a) XRD 2 theta-omega scans around the (0002) peak for the single ZnO and MgZnO films and MgZnO/ZnO bilayer. (b) AFM image of a typical area of the ZnO film which defines the MgZnO/ZnO interface. RMS roughness was < 1 nm over an area of 5 × 5 μm^2.

Fig. S1a shows XRD 2 theta-omega scans for the (0002) peak of the ZnO/sapphire, MgZnO/sapphire and MgZnO/ZnO/sapphire samples. For the ZnO/sapphire, the 2 theta-omega peak position corresponds to a *c*-lattice parameter of 5.218 Å (as compared to an equilibrium value of about 5.206 Å). This suggests that the film was under compressive strain in the *a*-*b* plane. The scan also shows Pendellosung fringes, which indicate that the surface was relatively smooth over the scale of the diffraction spot (few mm^2). The fringe spacing gives an estimate of film thickness at about 110 nm. The MgZnO/sapphire peak position indicates a *c*-lattice parameter of 5.202 Å. This is close to the ZnO equilibrium value, which is consistent since the substitutional Mg^{2+} ion has a fairly similar ionic radius to the Zn^{2+} ion[1]. There are no Pendellosung fringes in this case, which implies that the MgZnO surface morphology is rougher than that for the ZnO layer. The MgZnO/ZnO/sapphire peak position corresponds to a *c*-lattice parameter of 5.216 Å, which is significantly larger than the value found for MgZnO/sapphire and similar to that for the ZnO/sapphire. This suggests that there has been epitaxy on the ZnO underlayer and that the MgZnO overlayer is also strained. Once again, there are no Pendellosung fringes, thus there appears to be degradation in surface morphology compared to the underlying ZnO surface. The sample is thicker, so some suppression of the fringes can be expected independent of surface quality. The asymmetrical form of the (0002) 2theta-omega peaks for the

ZnO and MgZnO layers grown directly on sapphire indicate that there is strain relaxation in the both layers during growth. Asymmetry is also observed for the 2theta-omega scan of the MgZnO layer grown on the ZnO/sapphire; however, the MgZnO and ZnO peaks are superposed so it is difficult to distinguish if the asymmetry is present in both the MgZnO and ZnO layers or just the ZnO layers. Based on the small expected lattice mismatch between MgZnO and ZnO[2], however, we can expect relatively low strain in the MgZnO overlayer.

The XRD omega-scan rocking curve linewidths (high resolution optics) are 0.006º, 0.15º and 0.06º, respectively, for the ZnO, MgZnO and MgZnO/ZnO. This indicates that the dispersion in the crystallographic orientation about the *c*-axis was higher for the MgZnO samples compared to the ZnO. The MgZnO layer grown on the ZnO underlayer, however, was significantly more *c*-axis oriented than the MgZnO layer grown directly on sapphire. Overall, the XRD results indicate that incorporation of Mg generally degrades the crystal quality compared to ZnO itself (as reported elsewhere[3]) but that use of a ZnO underlayer considerably improves the MgZnO layer quality.

Fig. S1b shows an AFM image of a typical area of the ZnO film grown on *c*-Al_2O_3. AFM analysis revealed a root mean square (RMS) roughness of less than 1 nm over an area of 5 µm × 5 µm) for the ZnO underlayer.

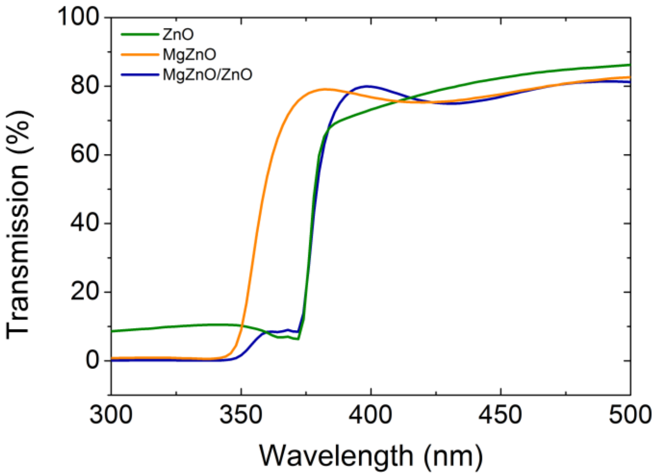

Figure S2. Transmission spectra for the single-layer ZnO and MgZnO films and the MgZnO/ZnO bilayer at room temperature. The absorption edges correspond to E_g(ZnO) = 3.34 eV and E_g(MgZnO) = 3.61 eV.

2. Depth-resolved cathodoluminescence microanalysis

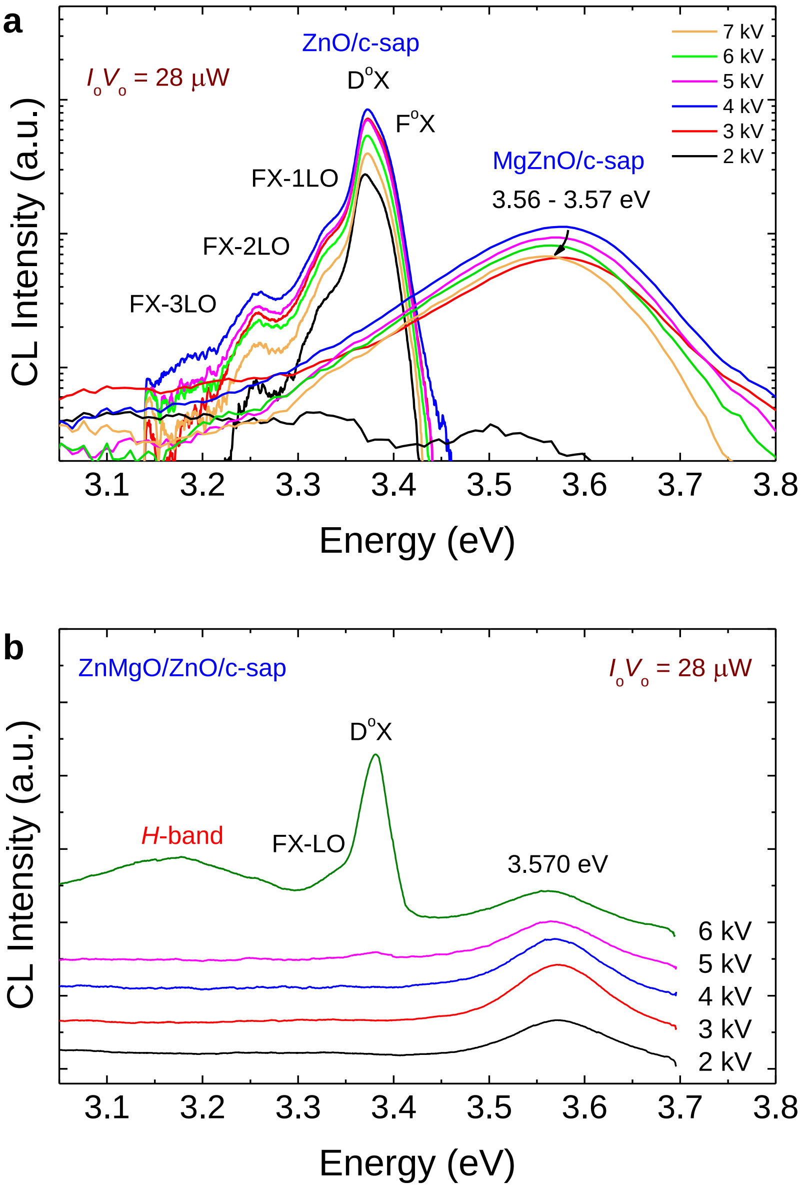

Figure S3. Depth-resolved CL spectra acquired with constant beam power for (a) single ZnO/c-sapphire and MgZnO/c-sapphire films and (b) the MgZnO/ZnO/c-sapphire bilayer acquired with various acceleration voltages between 2 and 7 kV. The shape of the CL spectra of ZnO/c-sapphire does not change with accelerating voltage, but the CL intensity increases with acceleration voltage due to suppression of non-radiative surface recombination. The emission of the single MgZnO film is slightly red-shifted, compared with the MgZnO in the bilayer, suggesting the tensile strain between MgZnO and the *c*-sapphire substrate[4]. For the bilayer there

is a large increase in the emission intensity for the bilayer as the beam energy is increased from 5 kV to 6 kV. This increase is consistent with the simulated electron energy loss curves displayed in Figure 3, which shows that the primary electron beam starts to reach the MgZnO/ZnO interface at 5 kV and that the energy deposition in the ZnO layer increases rapidly from this acceleration voltage.

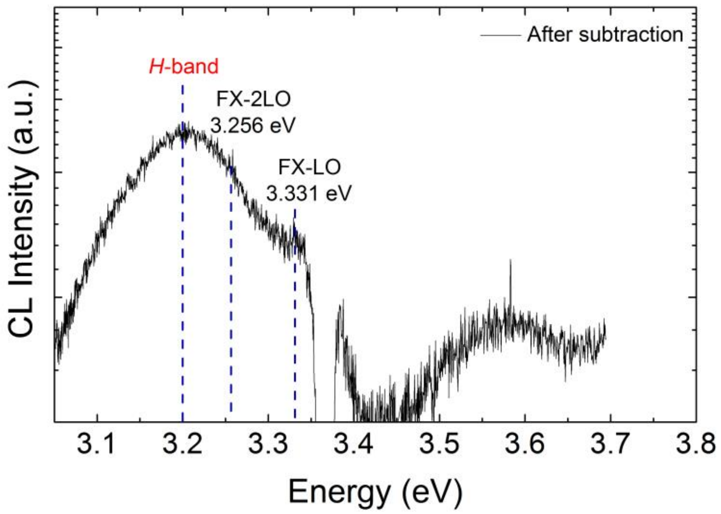

Figure S4. The interface *H*-band from the MgZnO/ZnO bilayer centred at 3.2 eV at 80 K, obtained by subtracting the ZnO emission contribution from the bilayer spectrum after normalization to the D^0X peak. Also shown in the spectrum are the energetic positions of phonon replicas of free excitons (FX-LO and FX-2LO).

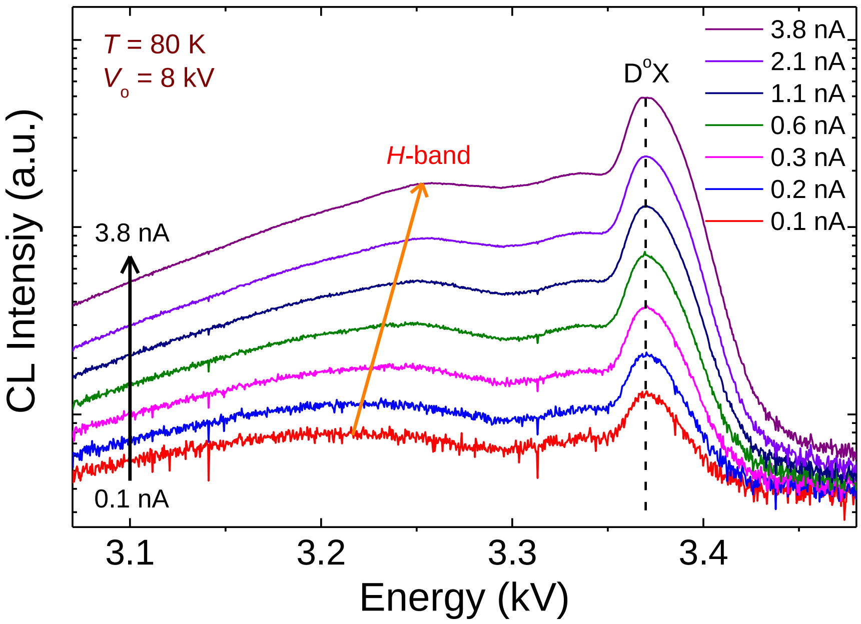

Figure S5. Power-dependent CL spectra of MgZnO/ZnO/c-sapphire bilayer structure. With increasing the excitation power, the H-band is blue shifted by ~ 40 meV. This is likely caused by the band bending field being screened by excess carriers that are generated by the electron beam.

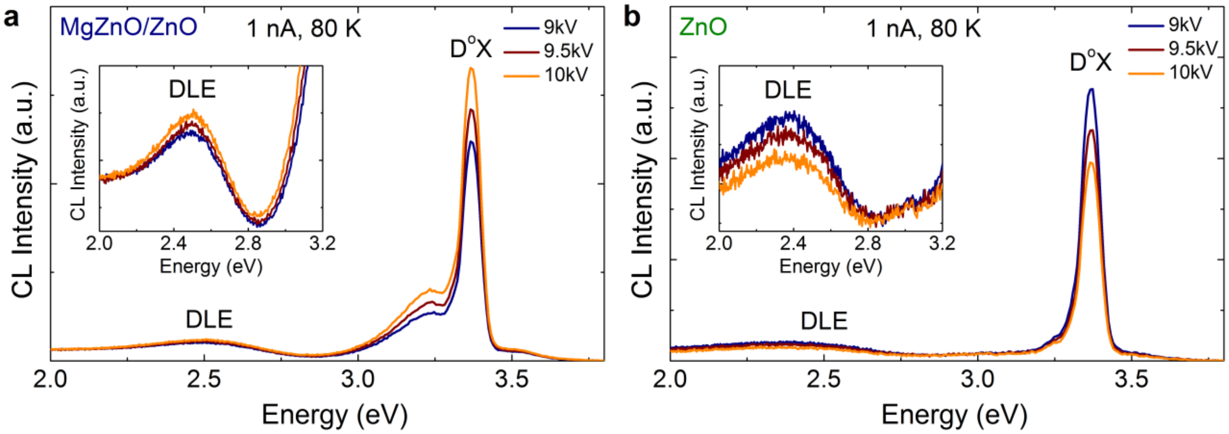

Figure S6. Depth resolved CL spectra analysis of defect-related deep-level emission (DLE) over the acceleration voltage range where the beam energy deposition at the interface is maximum for (a) MgZnO/ZnO bilayer and (b) single ZnO film. The intensity ratio of the DLE to excitonic D^oX emission remains at 0.70 ± 0.03 for both the bilayer and single layer film, indicating that no additional deep-level recombination centers are formed at the MgZnO/ZnO interface.

References

1 Takahashi, N., Makino M., Nakamura T. & Yamamoto H. Rapid growth of thick ZnO films with room-temperature ultraviolet emission by means of atmospheric pressure halide vapor-phase epitaxy. *Chem. Mater.* **14,** 3622-3624 (2002).

2 Liu, B. et al. Surface roughness scattering in two dimensional electron gas channel. *Appl. Phys. Lett.* **97,** 262111 (2010).

3 Lorenz, M. et al. Optical and electrical properties of epitaxial $(Mg, Cd)_xZn_{1-x}O$, ZnO, and ZnO:(Ga, Al) thin films on c-plane sapphire grown by pulsed laser deposition. *Solid-State Electron.* **47,** 2205-2209 (2003).

4 Su, L. et al. Solar-blind wurtzite MgZnO alloy films stabilized by Be doping. *J. Phys. D: Appl. Phys.* **46,** 245103 (2013).
